# Insights into Cytotoxic Behavior of Lepadins and Structure Elucidation of the New Alkaloid Lepadin L from the Mediterranean Ascidian *Clavelina lepadiformis*

**DOI:** 10.3390/md20010065

**Published:** 2022-01-11

**Authors:** Marcello Casertano, Massimo Genovese, Paolo Paoli, Alice Santi, Anna Aiello, Marialuisa Menna, Concetta Imperatore

**Affiliations:** 1 Department of Pharmacy, University of Naples “Federico II”, Via D. Montesano 49, 80131 Naples, Italy; marcello.casertano@unina.it (M.C.); aiello@unina.it (A.A.); cimperat@unina.it (C.I.); 2 Department of Experimental and Clinical Biomedical Sciences, University of Florence, Viale Morgagni 50, 50134 Florence, Italy; m.genovese13@gmail.com (M.G.); paolo.paoli@unifi.it (P.P.); alice.santi@unifi.it (A.S.)

**Keywords:** marine natural products, ascidians, *Clavelina lepadiformis*, decahydroquinoline-based alkaloids, structural determination, cytotoxic activity

## Abstract

The chemical investigation of the Mediterranean ascidian *Clavelina lepadiformis* has led to the isolation of a new lepadin, named lepadin L, and two known metabolites belonging to the same family, lepadins A and B. The planar structure and relative configuration of the decahydroquinoline ring of lepadin L were established both by means of HR-ESIMS and by a detailed as extensive analysis of 1D and 2D NMR spectra. Moreover, microscale derivatization of the new alkaloid lepadin L was performed to assess the relative configuration of the functionalized alkyl side chain. Lepadins A, B, and L were tested for their cytotoxic activity on a panel of cancer cell lines (human melanoma [A375], human breast [MDA-MB-468], human colon adenocarcinoma [HT29], human colorectal carcinoma [HCT116], and mouse myoblast [C2C12]). Interestingly, a deeper investigation into the mechanism of action of the most cytotoxic metabolite, lepadin A, on the A375 cells has highlighted its ability to induce a strongly inhibition of cell migration, G2/M phase cell cycle arrest and a dose-dependent decrease of cell clonogenity, suggesting that it is able to impair self-renewing capacity of A375 cells.

## 1. Introduction

**Figure 1:**
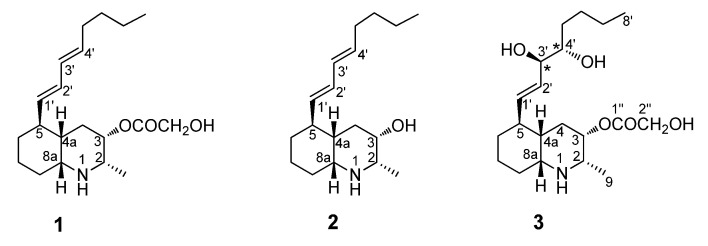
Chemical structures of lepadins A (**1**) and B (**2**), and of the new lepadin L (**3**).

The marine environment represents an extraordinary source of new and structurally complex bioactive metabolites characterized by a wide and incredible array of chemical structures showing antiallergic, anti-atherosclerotic, antibacterial, anticancer, anticoagulant, and antidiabetic to antifungal, antihyperlipidemic, antihypertensive, anti-inflammatory, and antioxidant to antiviral, cardioprotective, immunoadjuvant, and hypocholesterolemic activity [1,2]. Ascidians represent highly preferential drug resources in the ocean, being prolific producers of bioactive marine natural products, due to the strong immune defensive system and their associated symbiotic microbes [3,4,5]. In particular, the unique molecules isolated from ascidians have contributed with their considerable structural diversity, that remain a key inspirational factor in the search for new therapeutics, to a wealth of active compounds. Typically, most ascidian compounds contain one or two heteroatoms, most frequently nitrogen and/or oxygen and occasionally sulfur, that are presumably derived from amino acids and many of these are biologically active [6,7]. The lepadin family, comprising, until now, eleven decahydroquinoline members, was identified from ascidians belonging to the genera *Clavelina*, *Didemnum*, and *Aplidium* as well as their predators [8,9,10,11,12]. These secondary metabolites are characterized by 5-alkyl-3-hydroxydehydroquinoline nucleus with a diversified array of relative stereochemical relationships at C-2, C-3, C-4a, C-5, and C-8a (Figure 1). Lepadin alkaloids possess biological activities ranging mainly from cytotoxicity against cancer cell lines, inhibition of tyrosine kinase and butyrylcholine esterase activity, as antiparasitic properties [10,11]. Thus, lepadins may represent a promising class of marine natural products for the development of novel therapeutic agents. Alongside these interesting and varied pharmacological activities, the limited amounts of natural metabolites which can be isolated by complex screening procedures with the risk to have altered biological responses, as well as the hard issue to solve the definitive configurational assignments, have encouraged several researchers to undertake synthetic protocols for obtaining lepadins [13,14,15,16]. However, the synthesis of lepadins requires a conspicuous number of highly stereoselective synthetic steps which represents a not easily accessible route for obtaining the native metabolites. Indeed, only recently, Tong et al. have reported a more attractive synthetic strategy for constructing the common *cis*-fused decahydroquinoline ring of several lepadins by a green chemistry approach [17]. For this reason, the isolation of new examples of lepadin alkaloids remains a key factor to enlarge the great chemodiversity associated to this chemical class, and to provide further new structural motifs that can represent a valuable resource both to grow up the knowledge on their bioactivities and to assess accordingly structure-activity relationships.

In the frame of our research project aimed at the detailed chemical characterization of marine secondary metabolites from invertebrates [6,18,19], herein, we describe the results provided by the analysis of the metabolic content of the Mediterranean ascidian *Clavelina lepadiformis*. This analysis led to the isolation of the already known lepadins A (**1**) and B (**2**) [9,10] along with a new alkaloid belonging to the same family, named lepadin L (**3**, Figure 1). We have then explored their potential effects on viability and proliferation on different cancer cell lines, namely A375, MDA-MB-468, HT29, HCT116 cells, and C2C12 myoblasts; particularly, comparison of the pharmacological properties of compounds **1**–**3** allowed us to perform an assessment of simple structure–activity relationships which could be related to the different cytotoxicity.

## 2. Results and Discussion

### 2.1. Isolation of Lepadins A, B, and L and Structure Elucidation of Lepadin L

Several specimens of the colonial ascidian *C. lepadiformis*, collected in the bay of Pozzuoli (Naples) in 2018, were exhaustively extracted with methanol and, then, with chloroform. The concentrated extracts were combined, concentrated under reduced pressure, and partitioned between ethyl acetate and butanol. This procedure allowed to obtain three organic extracts with different polarity in which a primarily separation of compounds could be observed. Medium pressure flash chromatography of the ethyl acetate soluble material on a silica gel column, followed by repeated RP-18 high-performance liquid chromatography (HPLC) separations of the fractions eluted with EtOAc:MeOH 7:3, yielded the new lepadin L (**3**), together with the known compounds, lepadins A (**1**) and B (**2**) in the pure form. The known compounds **1** and **2** were readily identified by comparison of their physical and spectral data with those reported in the literature [9,10]. Despite the availability of only minute amounts of the new natural alkaloid **3**, its relative configuration assignment was realized. The stereochemistry elucidation of the pure compound lepadin L was performed mainly by a rigorous combination of different approaches which required mainly the *J* coupling analysis and 2D NOESY experiments, as well as required microscale chemical derivatization, too.

The HRESIMS of lepadin L (**3**) showed peaks at *m*/*z* 370.2585 [M + H]^+^ and at *m*/*z* 392.2403 [M + Na]^+^, suggesting the molecular formula C_20_H_35_NO_5_ with four degrees of unsaturation. The MS/MS fragmentation pattern of **3** was compatible with the presence of a 2-hydroxyacetic moiety, displaying the peak at *m*/*z* 294.2422 [M − C_2_H_4_O_3_ + H]^+^. The ^1^H NMR spectrum of **3** (Table 1) showed typical resonances of a decahydroquinoline core. The ^13^C NMR spectrum (Table 1) displayed signals for all 20 carbons, including an ester carbonyl carbon at *δ*_C_ 171.8. The general features of the proton spectrum of lepadin L (**3**) clearly resembled those of other lepadins, as illustrated by (i) the large band of alkyl chain methylene protons at *δ*_H_ 1.30, (ii) the diagnostic resonances of perhydroquinoline ring system (H-2, H-3, H-4a, H-8a), (iii) the typical oxymethine proton at *δ*_H_ 5.18, and (iv) a secondary aliphatic methyl group (*δ*_H_ 1.33, d, *J* = 6.0 Hz). The analysis of COSY, HSQC, and HMBC 2D NMR spectra of **3** led to the assignment of all protonic and carbon resonances through the key recorded homonuclear and heteronuclear correlations (Table 1). The 2D NMR spectra allowed to highlight a close correlation between the known lepadin A (**1**) and the novel lepadin L (**3**). Indeed, compounds **1** and **3** showed similar features due to the presence of the same decahydroquinoline core and the 2-hydroxyacetate moiety linked at C3. The most significant differences in the ^13^C NMR spectra of lepadin L and A were confined to the signals of the substituent at C-5 (C1′-C8′). Similarly, the ^1^H NMR spectra of these two compounds mainly differed for the resonances of two oxymethines of an acyclic 1,2 diol (*δ*_H_ 3.45 and *δ*_H_ 3.85). These data together with information provided by analysis of 2D NMR data allowed the sequential assignment of all the protons of the alkyl chain at C-5; indeed, ^1^H-^1^H spin system could be further extended to the side chain by following the chain of proton couplings from H-5 through to H-8′. The COSY spectrum evidenced H-5 to be coupled to a de-shielded methine proton resonating at *δ*_H_ 5.46 (H-1′, dd, *J* = 15.8 and 9.3 Hz), which is in turn coupled to a methine at *δ*_H_ 5.63 (H-2′, dd, *J* = 15.8 and 10.7 Hz). Furthermore, the latter proton was correlated to the oxymethine proton at δ_H_ 3.85 (H-3′, dd, *J* = 16.0 and 3.0 Hz) and this with another oxymethine proton resonating at *δ*_H_ 3.45 (H-4′, m), allowing us to locate the hydroxyl groups at C-3′, C-4′. Finally, the sequence was extended to the H-8′, taking advantage of HSQC and HMBC information.

**Figure 2:**
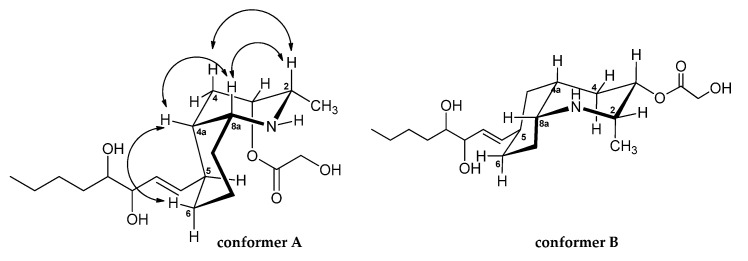
Possible chair-chair conformers (A and B) for the *cis*-azadecalin ring system of lepadin L. Key NOESY correlations (arrows) for the lepadin L (**3**).

The relative stereochemistry of lepadin L was established by a combination of 2D NOESY (Figure 2) experiment and the comprehensive analysis of the *J*_H-H_ coupling constant values. A diagnostic NOE cross peak between ^1^H NMR signals for H-4a and H-8a indicated a *cisoid* ring fusion of decahydroquinoline unit for **3**.

The *cis*-fused azadecalin ring system can exist in two chair–chair conformers [9,12]. The bicyclic ring conformation of **3** was established from NOESY experiments (Figure 1). In detail, the NOESY spectrum (Table 1 and Figure 2) of **3** contained key cross-peaks between signals for the paired 1,3-*syn* diaxial protons, H-2_ax_-H-4_ax_ and H-4a-H-6_ax_. These data are in agreement with the most stable conformation of *cis*-decahydroquinoline core in which the C-8a-N bond is situated axially with respect to the cyclohexane chair and with the side chain attached at C-5 equatorially oriented. It should be noted that this is also the favored conformation of *cis*-azadecalin ring systems in other lepadins.

The NOESY cross peaks between H-2_ax_ and H-8a showed these hydrogens adopted a diaxial disposition. The small coupling constant (^3^*J* = 2.4 Hz) between H-2 and H-3 revealed that they adopted a *syn* configuration and H-3 had to have an equatorial position, and the hydroxyacetyl group axially situated. By considering the information above, the protons H-2, H-3, H-4a, and H-8a were determined to be all *syn*-configured.

Moreover, scalar coupling of 15.8 Hz observed between H-1′ and H-2′ showed that the double bond had the *E* geometry.

**Scheme 1:**
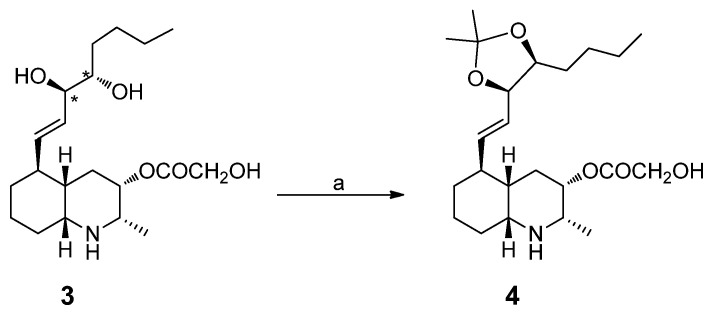
Conversion of lepadin L (**3**) into corresponding acetonide **4**. Reagents and conditions. (a) 2,2-dimethoxypropane, Dowex 50W-X8, rt, 2 h.

To establish the relative configuration at C-3′/C-4′, we prepared the acetonide derivative **4** by treating lepadin L (**3**) with 2,2-dimethoxypropane and a catalytic amount of Dowex 50W-X8 (Scheme 1). As is well known, the difference in the chemical shifts of the methyl groups in the dioxolane ring is always greater for the *cis*-acetonide (Δ*δ* 0.12–0.14), coming from the 1,2-*anti* isomer, when compared to the *trans*-acetonide (Δ*δ* 0.01–0.04), coming from the 1,2-*syn* isomer [20]. The observed Δ*δ* value of 0.11 ppm between the two methyl groups in **4** indicated the *cis* configuration for the dioxolane ring and, subsequently, the *anti* configuration of the 1,2-diol in lepadin L (**3**). 

### 2.2. In Vitro Evaluation of Cytotoxic Effects of Lepadins 1–3 on Different Cancer Cell Lines

**Figure 3:**
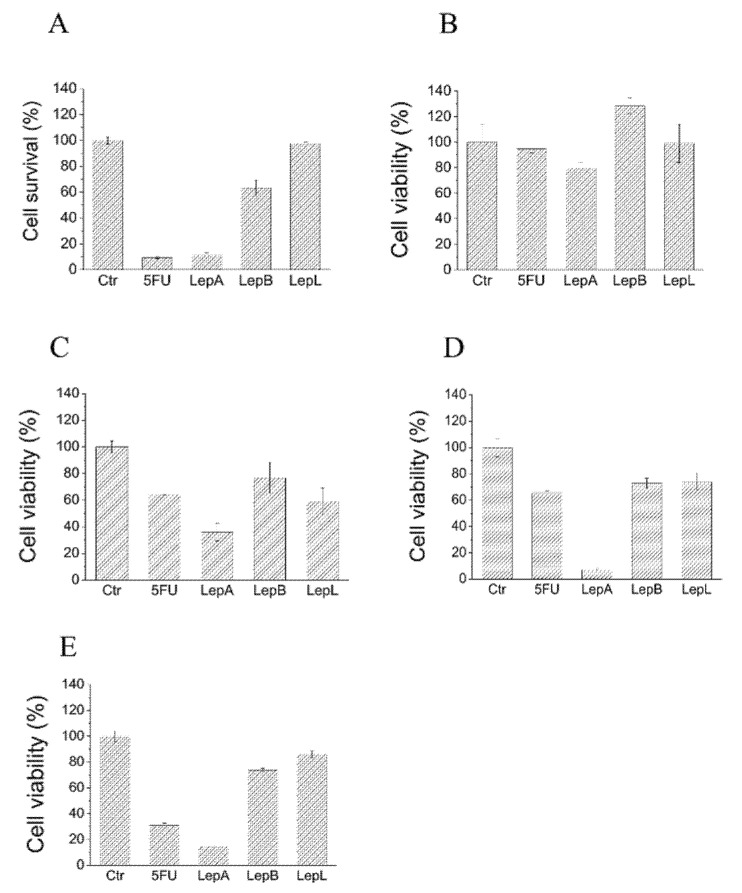
Evaluation of cytotoxic activity of lepadins **1**–**3** on different cancer cell lines. (**A**) A375; (**B**) MDA-MB-468; (**C**) HT29; (**D**) HCT116; and (**E**) C2C12 myoblasts. Cells were incubated in the presence of complete medium containing 50 µM lepadins or 100 µM 5-FU for 24 h at 37 °C. After this time, cell viability was determined using MTT assay. Each test was carried out in quadruplicate. Data showed in the figure represent mean value ± SD (*n* = 4). Ctr, control test carried out incubating cells in the presence of an equivalent volume of dimethyl sulfoxide, the solvent used to dissolve lepadins. 5FU, 5-Fluorouracil; LepA, lepadin A; LepB, lepadin B; and LepL, lepadin L.

The impact of lepadins A, B, and L (**1**–**3**) on the cell viability has been evaluated on a significant panel of cancer cell lines (human melanoma [A375], human breast [MDA-MB-468], human colon adenocarcinoma [HT29], human colorectal carcinoma [HCT116] and mouse myoblast [C2C12]) by using 5-fluorouracil (5-FU) as reference drug. The results are reported in Figure 3 where the cell survival percentages are graphically compared. As it can be observed, lepadin A was shown to be the most cytotoxic agent in the series; it exerted a strong cytotoxic effect toward A375, HCT116 human cells, and C2C12 mouse myoblasts (Figure 3, panels A–C), whereas it appeared moderately active towards HT29 cells, and completely inactive towards MDA-MB-468 cells (Figure 3, panels D and E). In contrast, lepadins B and L showed weak or no activity against the tested cancer cell lines (Figure 3, panels A–E).

### 2.3. Further Insights into the Cytotoxic Effects of Lepadin A on A375 Cells

**Figure 4:**
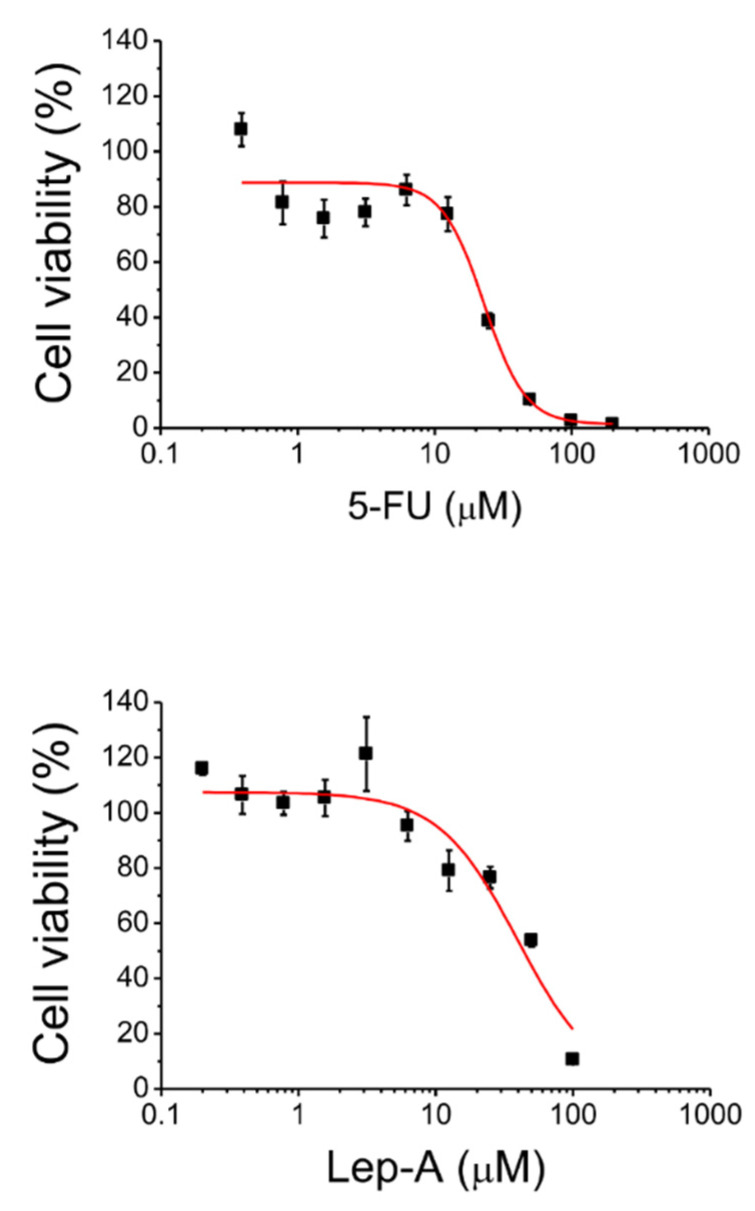
Determination of IC_50_ value of lepadin A (**1**) and 5-FU for A375 cells.

In recent years, the use of target therapy and immunotherapy drugs has made it possible to improve both the quality and duration of life of numerous cancer patients suffering from aggressive and advanced forms of cancer such as metastatic melanoma [21]. Despite the undoubted progress made, most of patients treated with these innovative drugs show a poor prognosis due to emergence of resistant clones of cancer cells that proliferate regardless of treatments. This finding suggests that the identification of new anticancer drugs to complement current anti-melanoma therapies represent one the greatest challenges in the coming years. For this reason, we decided to analyze the impact of lepadin A on A375 cells and the first experiment was that to determine the lethal dose 50 (IC_50_). To this aim, A375 cells were incubated in the presence of increasing concentrations of compound **1**; again, 5-FU has been used in this assay as positive control. The results showed by this assay confirmed the strong cytotoxicity exerted by lepadin A; accordingly, we have observed that compound **1** decreased cell survival in a dose-dependent manner (Figure 4), showing an IC_50_ value of 45.5 ± 4.0 µM, comparable to that obtained for the largely used anti-cancer chemotherapy drug 5-FU (IC_50_ = 22.9 ± 4.0 µM). Then, to evaluate the underlying mechanism of the highly significant effect of lepadin A on A375 cells viability, we have performed further in vitro experiments, as described below.

#### 2.3.1. Impact on Cell Migration

**Figure 5:**
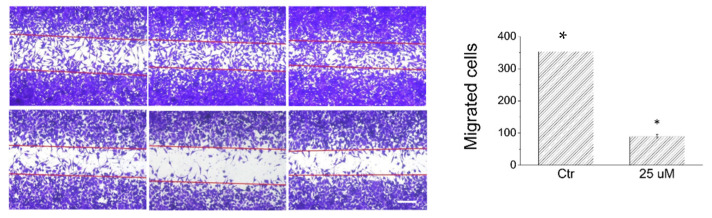
Wound-healing assay. The images were obtained using a contrast phase microscopy. Scale bar, 200 μm. The experiment was carried out in triplicate. For each well, various images derived from different regions were acquired. The data are presented as the mean ± SD (*n* = 3), * *p* < 0.005.

In the light of the interesting effects exerted by lepadin A on A375 cell line, we have evaluated the impact of this alkaloid on migration of A375 cells through a wound-healing assay. The wound-healing assay is a simple and inexpensive method to study directional cell migration in vitro, mimicking the cell migration during wound healing in vivo [22]. In our study, the plates containing confluent A375 cells have been scratched with a tip to generate a wound and then incubated for 16 h in the presence of complete medium supplemented or not with 25 µM of lepadin A. The images have been obtained using a contrast phase microscopy; the number of migrated cells has been determined by counting the cells moved into the wound. As shown in Figure 5, lepadin A decreases the cover rate of the scratch width and strongly inhibits cell migration.

#### 2.3.2. Cell Cycle Analysis

**Figure 6:**
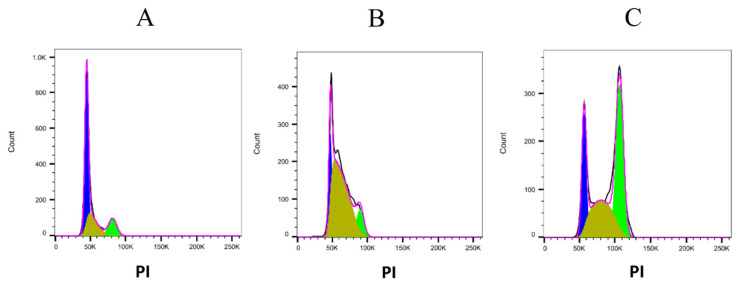
Representative pictures obtained after the cell cycle analysis performed using FlowJo software. (**A**) control cells; (**B**) A375 cells treated with 10 µM 5-FU; and (**C**) A375 cells treated with 40 µM lepadin A. PI, propidium iodide.

**Figure 7:**
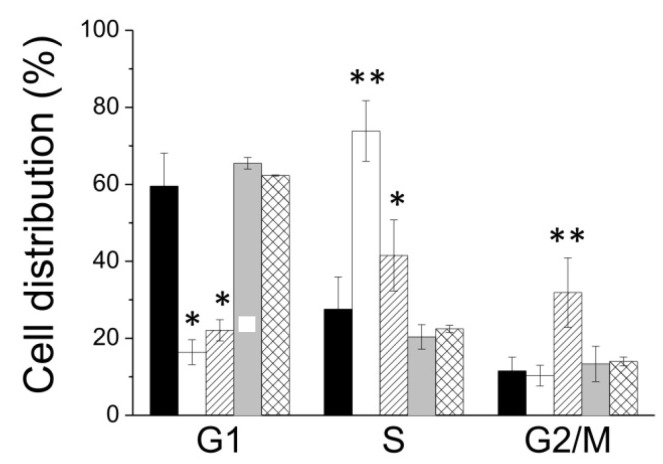
Distribution of cells into cell cycle phases express as percentage. Data showed in the figure represent the mean value ± SD. Each assay was performed in quadruplicate (*n* = 4). Black bars, control; white bars, A375 cells treated with 5-FU; and oblique lines, A375 cells treated with lepadin A 40 µM. * *p* < 0.05; ** *p* < 0.01.

Then, we have performed the cell cycle analysis by flow cytometer based on propidium iodide (PI) staining (Figure 6). A375 cells were incubated in the presence of 10 µM 5-FU, or 40 µM of lepadin A and, after 24 h, cells were washed and incubated in the presence of propidium iodide. The percentage of cells in the cell cycle phases was determined using a flow cytometer. The obtained results (Figure 7) showed that lepadin A induced G2/M phase cell cycle arrest in A375 cells at 40 µM. Figure 6A,B showed that the cell number in both S and G2/M phase increased in A375 lepadin A-treated cells suggesting that compound **1** interferes with S phase and leads to G2/M phase cell cycle arrest.

#### 2.3.3. Clonogenic Assay

**Figure 8:**
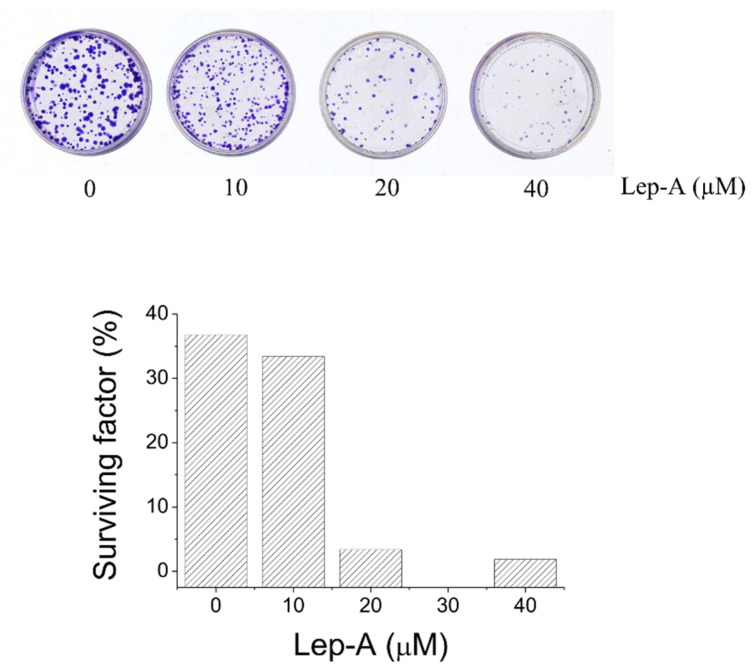
Clonogenity assay of A375 cancer cell line treated with increasing concentration of lepadin A (0, 10, 20, and 40 µM) for 24 h.

It is known that the self-renewal process of normal stem cells is deregulated in cancer stem cells resulting in a continuous expansion of self-renewing cancer cells and tumor formation. Therefore, agents targeting the defective self-renewal pathways in cancer cells might lead to improved outcomes in the treatment of cancer [23]. Thus, we have performed a clonogenic assay with the aim to investigate the ability of lepadin A to impair self-renewing capacity of melanoma A375 cells. To this purpose, growing A375 cells were incubated with increasing concentration (0, 10, 20, and 40 µM) of the natural alkaloid **1** for 24 h (Figure 8), and then detached, counted, and seeded in new wells (500 cells for each well). After 15 days, plates were stained with crystal violet to determine the number of colonies. Plating efficiency and surviving factor have been calculated as previously described [24]. These results demonstrate that lepadin A causes a dose-dependent decrease of cell clonogenity, suggesting that it is able to impair self-renewing capacity of A375 cells (Figure 8).

Herein, the whole reported data showed that lepadin A is able to impair viability and inhibit cell migration as well as self-renewal ability of A375 cells. Moreover, we found that A375 cells treated with lepadin A were significantly segregated into G2/M cell cycle phase, suggesting that it could interfere with tubulin assembling during mitotic spindle formation. Although this study is in its infancy in determining the mechanism of action of lepadin A, our data suggest that such kind of molecules could be used as lead compounds to generate a new class of microtubule-binding agents able to block proliferation of cancer cells. Further studies are ongoing to clarify the lepadin A mechanism of action as well as the activity of other lepadins on different kind of cancer cell lines.

## 3. Materials and Methods

### 3.1. General Experimental Procedures

High-resolution MS (positive mode) was performed on a Thermo LTQ Orbitrap XL mass spectrometer (Thermo-Fisher, San Josè, CA, USA). The spectra were recorded by infusion into the ESI source using MeOH as solvent. ^1^H NMR and 2D NMR experiments were carried out at 700 MHz on a Bruker Avance Neo spectrometer (Bruker BioSpin Corporation, Billerica, MA, USA); chemical shifts were referenced to the residual solvent signal (CD_3_OD: *δ*_H_ = 3.31, *δ*_C_ = 49.0). Two and three bond ^1^H-^13^C connectivities were determined by gradient 2D HMBC experiments optimized for a ^2,3^*J* of 8 Hz. Through-space ^1^H connectivities were evidenced using a NOESY experiment with a mixing time of 300 ms. A Jasco P-2000 polarimeter (Jasco Europe s.r.l., Cremella, Italy) at the sodium D line was used to measure optical rotations. High performance liquid chromatography (HPLC) separation was achieved on a Knauer K-501 apparatus equipped with a Knauer K-2301 RI detector (LabService Analytica s.r.l., Anzola dell’Emilia, Italy).

### 3.2. Collection, Extraction, and Isolation

Specimens of *C. lepadiformis* were collected in the bay of Pozzuoli (Napoli, Italy 40°49′39.61″ N 14°09′11.56″ E) in the spring of 2018 at a depth of approximatively 10 m, by hand, using scuba. They were frozen immediately after collection and stored at −20 °C until extraction. The taxonomical identification of the organisms was carried out by Mr. Arturo Facente. A voucher specimen is deposited at the Department of Pharmacy, University of Naples “Federico II”, Naples, Italy.

Fresh thawed animals (42.6 g dry weight after extraction) were homogenized and extracted with MeOH (3 × 400 mL) and then with CHCl_3_ (3 × 400 mL). Extracts were combined and concentrated in vacuo; the resulting aqueous residue was extracted with EtOAc and subsequently with n-BuOH. The ethyl acetate-soluble material was chromatographed by MPLC over a silica gel column followed using an increasing gradient elution (100% *n*-hexane→*n*-hexane:EtOAc 9:1→*n*-hexane:EtOAc 7:3→*n*-hexane:EtOAc 1:1→*n*-hexane:EtOAc 3:7→100% EtOAc→EtOAc:MeOH 9:1→EtOAc:MeOH 7:3→EtOAc:MeOH 1:1→EtOAc:MeOH 3:7→100% MeOH→100% CHCl_3_) to yield twelve fractions 1–24. The fractions 15 and 16 eluted with EtOAc:MeOH 7:3 (*v*/*v*) were further purified by HPLC. Fraction 15 was chromatographed by RP-HPLC Luna 3 μm C-18 column, MeOH:H_2_O (6:4) and 0.1% of trifluoroacetic acid and yielded in pure form lepadin A (**1**, t*_R_* 25.5 min, 3.3 mg). Fraction 16 was analyzed by HPLC on Luna 3 μm C-18 column, MeOH:H_2_O (7:3) and 0.1% of trifluoroacetic acid and afforded lepadin B (**2,** t*_R_* 11.3 min, 13.5 mg) in pure state and a fraction mainly composed of lepadin L (**3**, t*_R_* 6.1 min, 7.8 mg). This latter fraction has been further purified by HPLC on a RP-18 column (Luna 3 μm C-18), CH_3_CN:H_2_O (3:7) and 0.2% of trifluoroacetic acid, thus affording compound **3** (t*_R_* 4.1 min, 1.2 mg) as pure compound. The purity of each metabolite (**1**–**3**) has been determined by ^1^H NMR analysis.

*Lepadin A* (**1**): colorless oil; ${\left[\alpha \right]}_{D}^{20}$ −29.1 (c 0.001, CHCl_3_); ^1^H NMR spectrum (CD_3_OD) is reported in Supplementary Material (Figure S1); HRMS (ESI): *m*/*z* 336.2527 [M + H]^+^ (calcd. for C_20_H_34_O_3_N: 336.2533); *m*/*z* 358.2346 [M + Na]^+^ (calcd. for C_20_H_33_O_3_NNa: 358.2353) (Figure S2).
*Lepadin B* (**2**): colorless oil; ${\left[\alpha \right]}_{D}^{20}$ −47.3 (c 0.001, CHCl_3_); ^1^H NMR spectrum (CD_3_OD) is reported in Supplementary Material (Figure S3); HRMS (ESI): *m*/*z* 278.2484 [M + H]^+^ (calcd. for C_18_H_32_ON: 278.2478) (Figure S4).
*Lepadin L* (**3**): colorless oil; ${\left[\alpha \right]}_{D}^{20}$ −8.1 (c 0.001, CHCl_3_); ^1^H and ^13^C NMR data (CD_3_OD) are reported in Table 1; 2D NMR data, Figures S5–S6 and S9–S12; HRMS (ESI): *m*/*z* 370.2585 [M + H]^+^ (calcd. for C_20_H_36_O_5_N: 370.2588); *m*/*z* 392.2403 [M + Na]^+^ (calcd. for C_20_H_35_O_5_NNa 392.2407) (Figures S7 and S8).

### 3.3. Synthesis of Lepadin L Acetonide (4)

A solution of lepadin L (**3**, 0.5 mg) in 2,2-dimethoxypropane (500 µL) was treated with Dowex 50W-X8 (H^+^ form, 20 mg) in catalytic amount, and the mixture was stirred for 2 h at room temperature. After this time, the resin was removed by filtration and the solvent from the filtrate was dried in vacuo affording compound **4** (0.5 mg). Moreover, traces of the related derivative endowed with a hydroxyl group at C-3 instead of the 2-hydroxyacetate ester functionality was observed.

*Lepadin L acetonide* (**4**): colorless oil; ^1^H NMR spectrum (CD_3_OD) is reported in Supplementary Material (Figure S13); HRMS (ESI): *m*/*z* 410.2899 [M + H]^+^ (calcd. for C_23_H_40_O_5_N: 410.2901); *m*/*z* 432.2718 [M + Na]^+^ (calcd. for C_23_H_39_O_5_NNa 432.2720) (Figure S15). ^1^H NMR (700 MHz, CD_3_OD) δ_H_ 3.64 (H-2, overlapped, 1H), 5.18 (H-3, m, 1H), 1.87–2.31 (H-4ax, H-4eq, m, 2H), 1.81 (H-4a, overlapped, 1H), 2.14 (H-5, m, 1H), 1.77–2.08 (H-6ax, H-6eq, overlapped, 2H), 1.75 (H-7ax, overlapped, 1H), 1.24 (H-7eq, m, 1H), 1.30–2.05 (H-8ax, H-8eq, overlapped, 2H), 3.57 (H-8a, overlapped, 1H), 1.33 (H-9, d, *J* = 7 Hz, 3H), 5.49 (H-1′, m, 1H), 5.39 (H-2′, m, 1H), 4.45 (H-3′, m, 1H), 4.43 (H-4′, m, 1H), 1.38 (H-5′, overlapped, 2H), 1.31 (H-6′, overlapped, 2H), 1.35 (H-7′, overlapped, 2H), 0.91 (H-8′, t, *J* = 14 Hz, 3H), 4.22 (H-2′’, s, 2H), 1.32 (H-1‴, s, 3H), 1.43 (H-3‴, s, 3H). ^13^C NMR (175 MHz, CD_3_OD) δ_C_ 56.2 (C-2, CH), 69.7 (C-3, CH), 29.5 (C-4, CH_2_), 37.1 (C-4a, CH2), 39.7 (C-5, CH), 29.1 (C-6, CH_2_), 34.0 (C-7, CH_2_), 29.3 (C-8, CH_2_), 58.5 (C-8a, CH), 14.7 (C-9, CH_3_), 127.1 (C-1′, CH), 139.3 (C-2′, CH), 81.1 (C-3′, CH), 81.1 (C-4′, CH), 33.9 (C-5′, CH_2_), 31.0 (C-6′, CH_2_), 23.4 (C-7′, CH_2_), 14.1 (C-8′, CH_3_), 171.8 (C-1′’, CO), 61.3 (C-2′’, CH_2_), 25.8 (C-1‴, CH_3_), 107.5 (C-2‴, C), 28.5 (C-3‴, CH_3_).

### 3.4. Cell Culture

The human cell lines (A375, MDA-MB-468, HT29, HCT116, and C2C12) were obtained from the American Type Culture Collection (Manassas, VA, USA). All cell lines were cultured in 1640 medium supplemented with 10% FBS and a 1% P/S solution. LS174T cells were cultured in Dulbecco’s modified Eagle’s medium (DMEM) containing 10% (*v*/*v*) foetal bovine serum (FBS; Gibco, Waltham, MA, USA) and a 1% (*v*/*v*) penicillin–streptomycin (P/S) solution (Gibco). HCT116 and HT29 cells were cultured in McCoy’s 5°medium supplemented with 10% FBS and a 1% P/S solution. SW480 and SW620 cells were cultured in Leibovitz’s L-15 medium supplemented with 10% FBS and a 1% P/S solution. All the cells were cultured at 37 °C in a humidified atmosphere with 5% CO_2_.

### 3.5. Cell Viability Assay

For the initial screening, cancer cells (1.5 × 10^4^/well) were seeded into 24-well plates and treated with lepadins A, B, and L (**1**–**3**) or 100 mM of 5FU for 24 h. Subsequently, medium was removed from well, cells were washed with PBS and then incubated at 37 °C in the presence of complete cell growth medium containing 0.5 mg/mL of MTT reagent. After 1 h, the medium was removed, the wells were washed with PBS before adding DMSO to lyse the cells and dissolve the formazan crystals. Cell viability was assessed by determination of the optical density (OD) at 595 nm using a microplate reader (550 Microplate Reader, Bio Rad CA, USA).

### 3.6. Clonogenic Assay

Melanoma cells were incubated with increasing concentration of lepadin A for 24 h and then tripsinized. Cells were counted and seeded on P35 dishes (500 cells for each plate). After 15 days, plates were incubated for 5 min at 37 °C in the presence of a crystal violet solution (0.2% w/v dissolved in a solution containing 20% methanol) to stain the colonies grown in each plate. The influence of the compounds on the clonogenicity was expressed as percent of control according to the formula: number of colonies generated after treatment with the compounds/number of colonies in control with the vehicle.

### 3.7. Cell Cycle Analysis

A375 cells (60.000 cells) were plated on P35 wells and incubated in the presence of 40 µM lepadins A, B, and L, or 10 µM of 5-FU for 24 h. Then, cell plates were washed with PBS and incubated in the presence of 50 µg/mL of propidium iodide (PI) dissolved in 0.1% Na-citrate, containing 0.1% NP40. Cell plates were stored in the dark for 15 min to allow cell lysis and the staining of nuclei. After this time, cell nuclei were collected and analyzed using a flow cytometer (FACS CANTO, BD Bioscience Franklin Lakes, NJ, USA). The distribution of cells into cycle phases was calculated using FlowJo software (FlowJo, LLC, Ashland, OR, USA).

### 3.8. Statistical Analysis

The results were expressed as mean ± SD. The differences between the experimental and control groups were compared using one-way ANOVA followed by Dunnett’s multiple comparisons test. All statistical analyses were performed using OriginPro 8.0 2021 software. The *p* value < 0.05 was considered statistically significant. * *p* < 0.05; ** *p* < 0.01.

## 4. Conclusions

An exhaustive examination of the chemical constituents of the Mediterranean ascidian *Clavelina lepadiformis* allowed us to expand the knowledge on the alkaloid composition of this marine organism with the addition of a new decahydroquinoline-based compound named lepadin L (**3**). The three isolated alkaloids, the known lepadins A and B (**1** and **2**), as well as the new lepadin L (**3**), were evaluated for their antiproliferative activity against a panel of cancer cell lines (A375, MDA-MB-468, HT29, HCT116 cells, and C2C12 myoblasts). Our pharmacological studies demonstrated that lepadin A behaved as strong cytotoxic agent toward A375, HCT116 cells, and C2C12 myoblasts, whereas lepadins B and L showed weak or no activity against all the tested cancer cell lines. Lepadin A strongly inhibits migration of A375 cells and interferes with S phase inducing G2/M phase cell cycle arrest in this cell line. Furthermore, the result of clonogenity assay highlighted compound **1** inhibits self-renewal capability of A375 cells. Together, these results suggested that lepadin A behaves as an antitumoral agent able to block cells cycle, cell migration, cell renewal ability, and to induce cell death. These findings agree with previous results showing that lepadin A, and to lesser extent lepadin B, was cytotoxic against several tumoral cell lines [10]. However, data we reported give important news about the antitumoral activity of these natural compounds and pave the way to use such molecules as lead compound for development of new potent antitumoral drugs. Further studies will be performed in the coming years in order to identify physiological targets of lepadin A, to deeply investigate its mechanism of action and in vivo effectiveness. The isolation of the new compound lepadin L, a metabolite closely related to the well-known lepadin A, as well as the results of our in-depth studies have allowed us to highlight important structural requirements for the cytotoxic activity of these natural compounds. In particular, it has been highlighted that the presence of two hydroxyl groups instead of a double bond on the substituent at C-5 in compound **3** causes a dramatic decrease of cytotoxicity and that, therefore, the polarity of these compounds is crucial for the fulfillment of the cytotoxic activity. These results further confirm that marine ascidians constitute an immense source of chemodiversity that can be beneficially exploited to discover new chemical entities for drug development.

## Figures and Tables

**Table 1:** ^1^H (700 MHz) e ^13^C (175 MHz) NMR data of compound **3** in CD_3_OD ^a^.

Pos.	*δ*_H_, Mult. (*J* in Hz)	*δ*_C_ (mult.)	NOESY	COSY	HMBC
**2**	3.63 ^b^	56.5 (CH)	3, 4_ax_, 4a, 8a, 8_eq_	3, 9	9
**3**	5.18, bs	69.5 (CH)	2, 4_ax_, 9	2, 4_ax_, 4_eq_	1″, 4a
**4_ax_**	1.87, m	29.5 (CH_2_)	2, 8a, 4_eq_	3, 4_eq_	3, 4a, 5, 8a
**4_eq_**	2.31, d (15.6)		4_ax_, 6_ax_	3, 4_ax_	2, 3, 4a, 5
**4a**	1.81, m	37.1 (CH)	2, 6_ax_, 8a	3, 5	4, 5, 68a, 1′
**5**	2.48, m	40.6 (CH)	6_ax_, 7_eq_, 1′, 2′	4a, 1′	4a, 1′
**6_ax_**	1.79, qd (13.3, 3.5)	29.2 (CH_2_)	4_eq_, 5, 7_eq_, 8a, 1′	5, 6_eq_, 7_ax_, 7_eq_	4a, 5, 7, 8
**6_eq_**	2.06, m		6_ax_, 7_ax_	6_ax_, 7_ax_, 7_eq_	4a, 7, 8
**7_ax_**	1.76, qt (13.3, 3.3)	34.1 (CH_2_)	6_ax_, 6eq, 8a, 8_eq_	6_eq_, 6_ax_,7_eq_, 8_ax_, 8_eq_	5, 6, 8a
**7_eq_**	1.24, m		6_ax_	6_eq_, 7_ax_	5, 8
**8_ax_**	1.70 ^b^	19.8 (CH_2_)	6_ax_	7, 8_a_, 8_eq_	6, 7, 8_a_
**8_eq_**	1.70 ^b^		7_ax_, 8a	7, 8_a_, 8_ax_	6, 7, 8_a_
**8a**	3.60 ^b^	58.6 (CH)	2, 4_ax_, 4a, 8_eq_	4_a_, 8_ax_, 8_eq_	7
**9**	1.33, d (6.0)	14.7 (CH_3_)	2, 3, 2″	2	3
**1′**	5.46, dd (15.8, 9.3)	136.1 (CH)	6_ax_, 3′, 5	5, 2′	5, 2′, 3′
**2′**	5.63, dd (15.8, 10.7)	132.3 (CH)	3′, 5	5, 3′	5, 1′, 3′, 4′
**3′**	3.85, dd (16.0, 3.0)	76.5 (CH)	4′	2′, 4′	1′, 2′, 4′, 5′
**4′**	3.45, m	75.4 (CH)	3′	3′, 5′	2′
**5′**	1.31 ^b^	33.1 (CH_2_)		4′, 6′	4′
**6′**	1.30 ^b^	31.3 (CH_2_)		5′, 7′	7′
**7′**	1.35, m	23.4 (CH_2_)		6′, 8′	6′, 8′
**8′**	0.91, t (7.0)	14.1 (CH_3_)		7′	6′, 7′
**1″**	-	171.8 (CO)			
**2″**	4.30, s	61.0 (CH_2_)	9		1″

^a 1^H NMR and ^13^C NMR shifts are referenced to CD_3_OD (*δ*_H_ = 3.31 ppm and *δ*_C_ = 49.0 ppm). The proton and carbon resonances were assigned by HSQC and HMBC experiments. ^b^ Partially overlapped to other ^1^H resonances.

## Data Availability

Not applicable.

## Supplementary Materials

The following are available online at https://www.mdpi.com/article/10.3390/md20010065/s1: Figure S1. ^1^H NMR spectrum in CD_3_OD (700 MHz) of lepadin A (**1**); Figure S2. HR-ESIMS spectrum of lepadin A (**1**); Figure S3. ^1^H NMR spectrum in CD_3_OD (700 MHz) of lepadin B (**2**); Figure S4. HR-ESIMS spectrum of lepadin B (**2**); Figure S5. ^1^H NMR spectrum in CD_3_OD (700 MHz) of lepadin L (**3**); Figure S6. ^13^C NMR spectrum in CD_3_OD (175 MHz) of lepadin L (**3**); Figure S7. HR-ESIMS spectrum of lepadin L (**3**); Figure S8. HR-ESI MS/MS spectrum of lepadin L (**3**); Figure S9. ^1^H-^1^H COSY NMR spectrum in CD_3_OD (700 MHz) of lepadin L (**3**); Figure S10. ^1^H-^13^C HSQC NMR spectrum in CD_3_OD (700 MHz) of lepadin L (**3**); Figure S11. ^1^H-^13^C HMBC NMR spectrum in CD_3_OD (700 MHz) of lepadin L (**3**); Figure S12. ^1^H-^1^H NOESY NMR spectrum in CD_3_OD (700 MHz) of lepadin L (**3**); Figure S13. ^1^H NMR spectrum in CD_3_OD (700 MHz) of lepadin L acetonide (**4**); Figure S14. Enlargement of ^1^H-^13^C-HMBC spectrum in CD_3_OD (700 MHz) of lepadin L acetonide (**4**); and Figure S15. HR-ESIMS spectrum of lepadin L acetonide (**4**).
