# Supplementary material for: Insights into Cytotoxic Behavior of Lepadins and Structure Elucidation of the New Alkaloid Lepadin L from the Mediterranean Ascidian Clavelina lepadiformis
Source: Mar Drugs. 2022 Jan 11;20(1):65. doi: 10.3390/md20010065 (PMC8782007; doi:10.3390/md20010065)

SUPPORTING INFORMATION

Figure S1. ^{1}H NMR spectrum in CD_3OD (700 MHz) of lepadin A (**1**) 1

Figure S2. HR-ESIMS spectrum of lepadin A (**1**) 1

Figure S3.^{1}H NMR spectrum in CD_3OD (700 MHz) of lepadin B (**2**) 2

Figure S4. HR-ESIMS spectrum of lepadin B (**2**) 2

Figure S5. ^{1}H NMR spectrum in CD_3OD (700 MHz) of lepadin L (**3**) 3

Figure S6. ^{13}C NMR spectrum in CD_3OD (175 MHz) of lepadin L (**3**) 3

Figure S7. HR-ESIMS spectrum of lepadin L (**3**) 4

Figure S8. HR-ESI MS/MS spectrum of lepadin L (**3**) 4

Figure S9. ^{1}H-^{1}H COSY NMR spectrum in CD_3OD (700 MHz) of lepadin L (**3**) 5

Figure S10. ^{1}H-^{13}C HSQC NMR spectrum in CD_3OD (700 MHz) of lepadin L (**3**) 5

Figure S11. ^{1}H-^{13}C HMBC NMR spectrum in CD_3OD (700 MHz) of lepadin L (**3**) 6

Figure S12. ^{1}H-^{1}H NOESY NMR spectrum in CD_3OD (700 MHz) of lepadin L (**3**) 6

Figure S13. ^{1}H NMR spectrum in CD_3OD (700 MHz) of lepadin L acetonide (**4**) 7

Figure S14. Enlargement of ^{1}H-^{13}C-HMBC spectrum in CD_3OD (700 MHz) of lepadin L acetonide (**4**) 7

Figure S15. HR-ESIMS spectrum of lepadin L acetonide (**4**) 8

Figure S1. ^{1}H NMR spectrum in CD_3OD (700 MHz) of lepadin A (**1**)

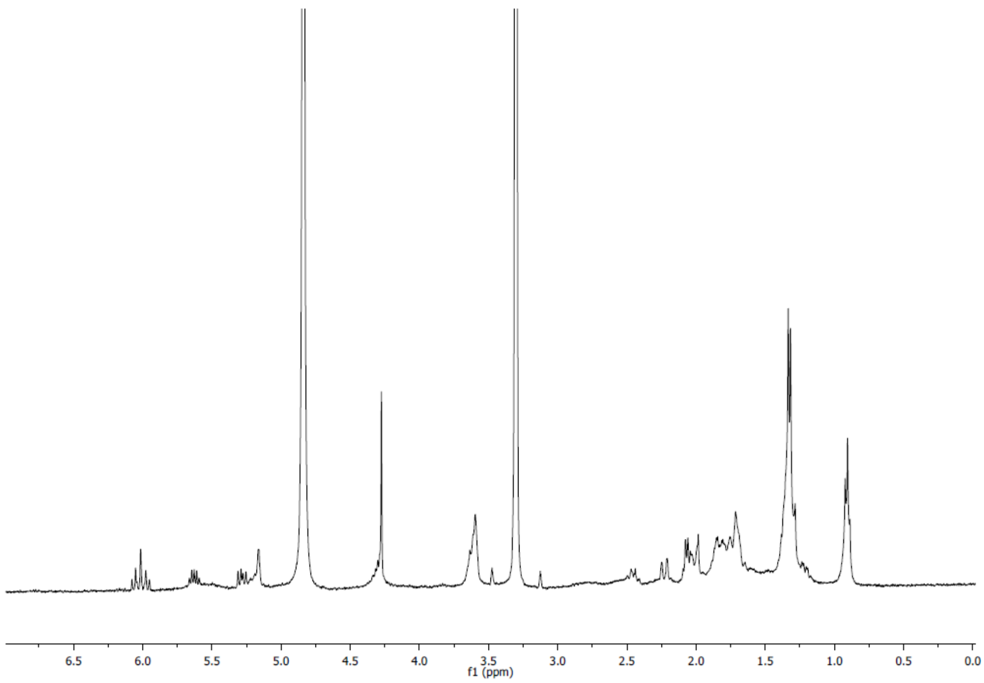

Figure S2. HR-ESIMS spectrum of lepadin A (**1**)

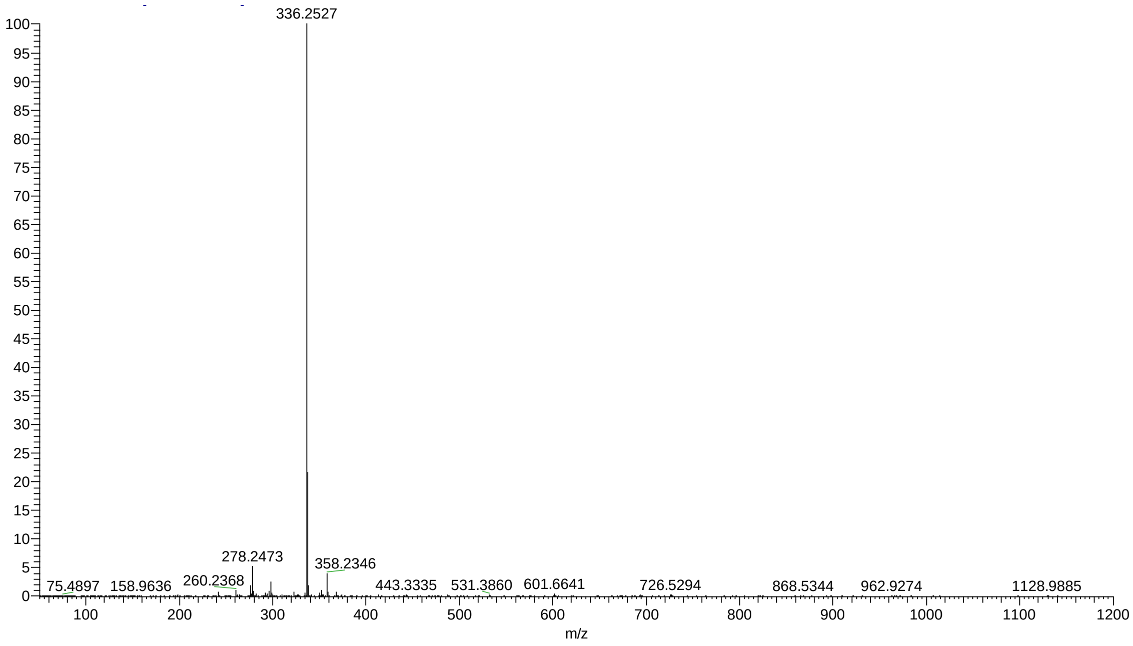

Figure S3. ^{1}H NMR spectrum in CD_3OD (700 MHz) of lepadin B (**2**)

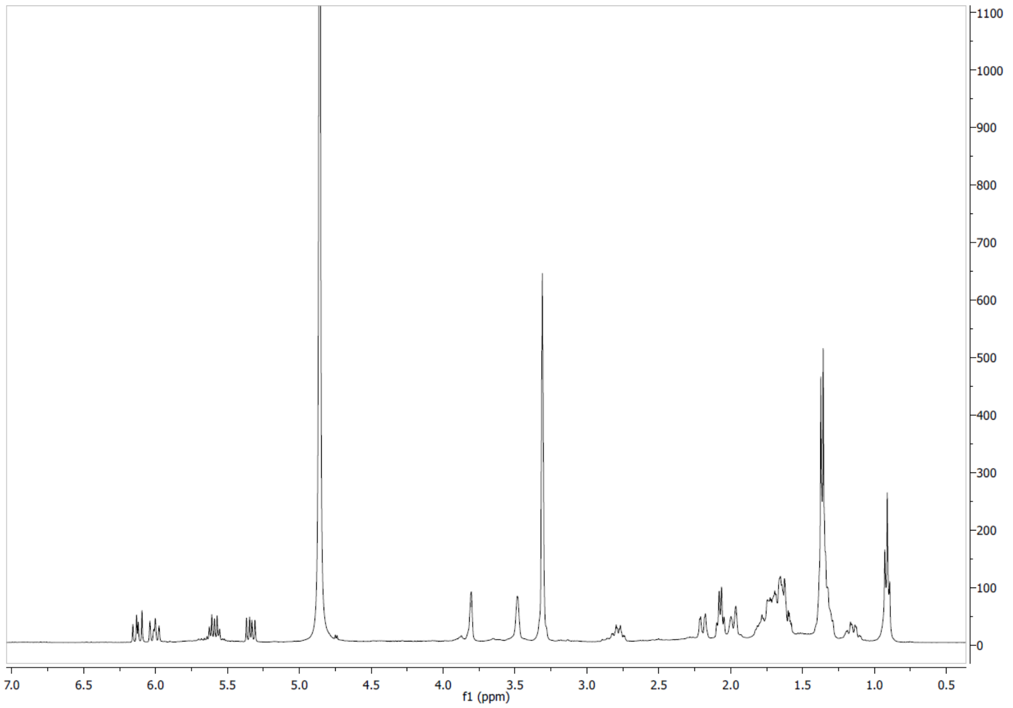

Figure S4. HR-ESIMS spectrum of lepadin B (**2**)

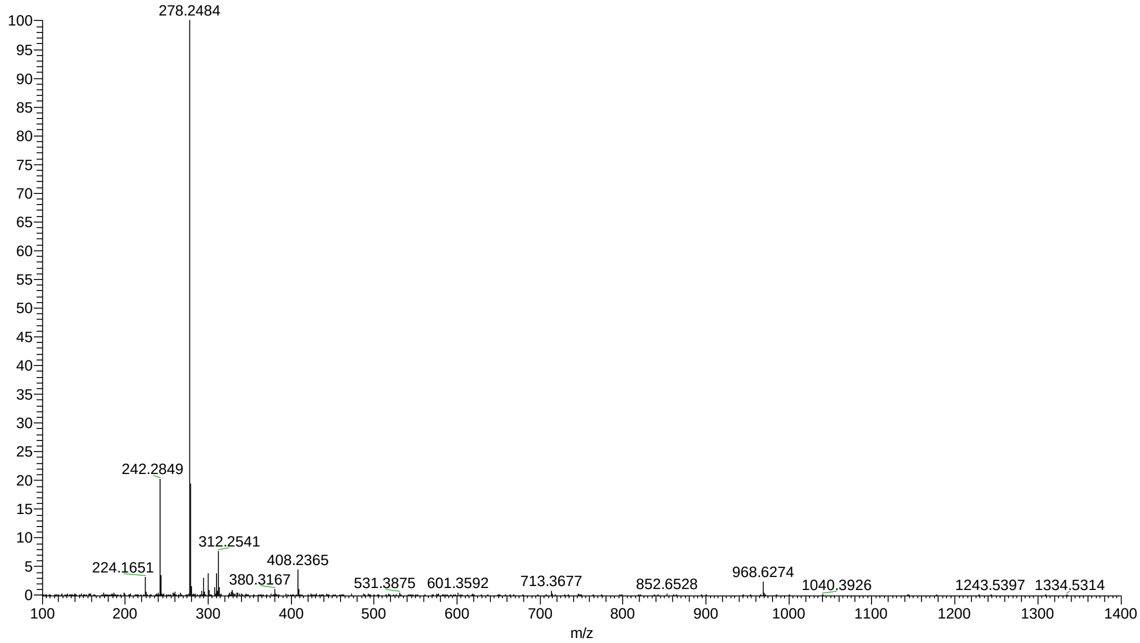

Figure S5. ^{1}H NMR spectrum in CD_3OD (700 MHz) of lepadin L (**3**)

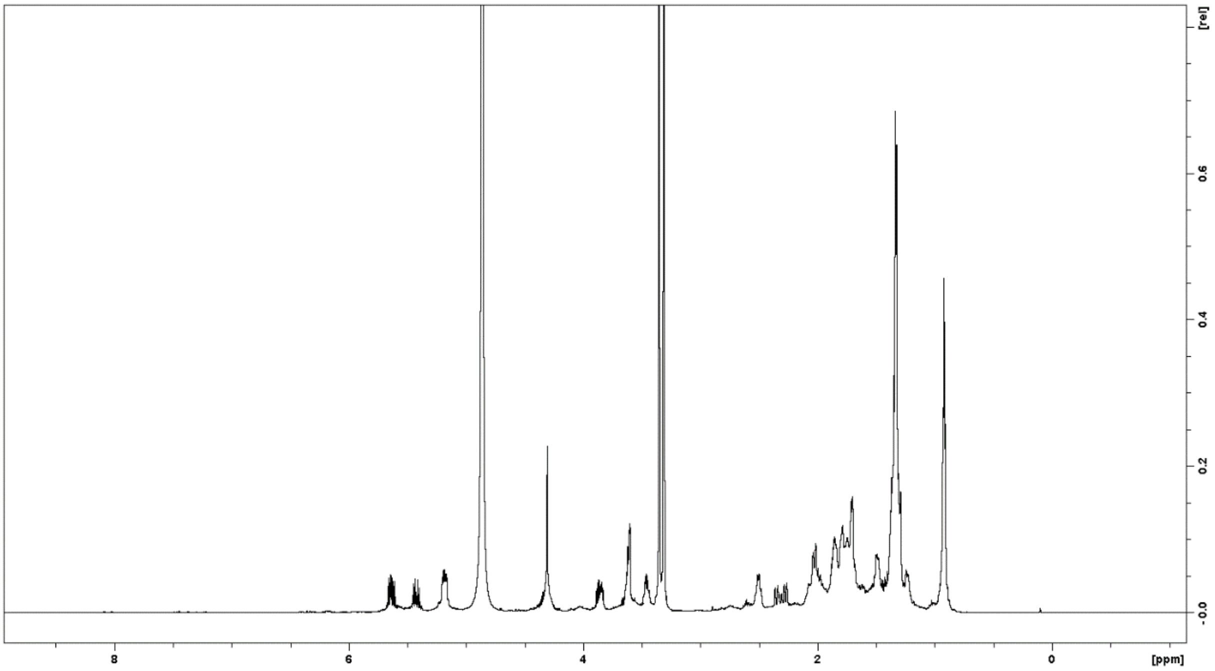

Figure S6. ^{13}C NMR spectrum in CD_3OD (175 MHz) of lepadin L (**3**)

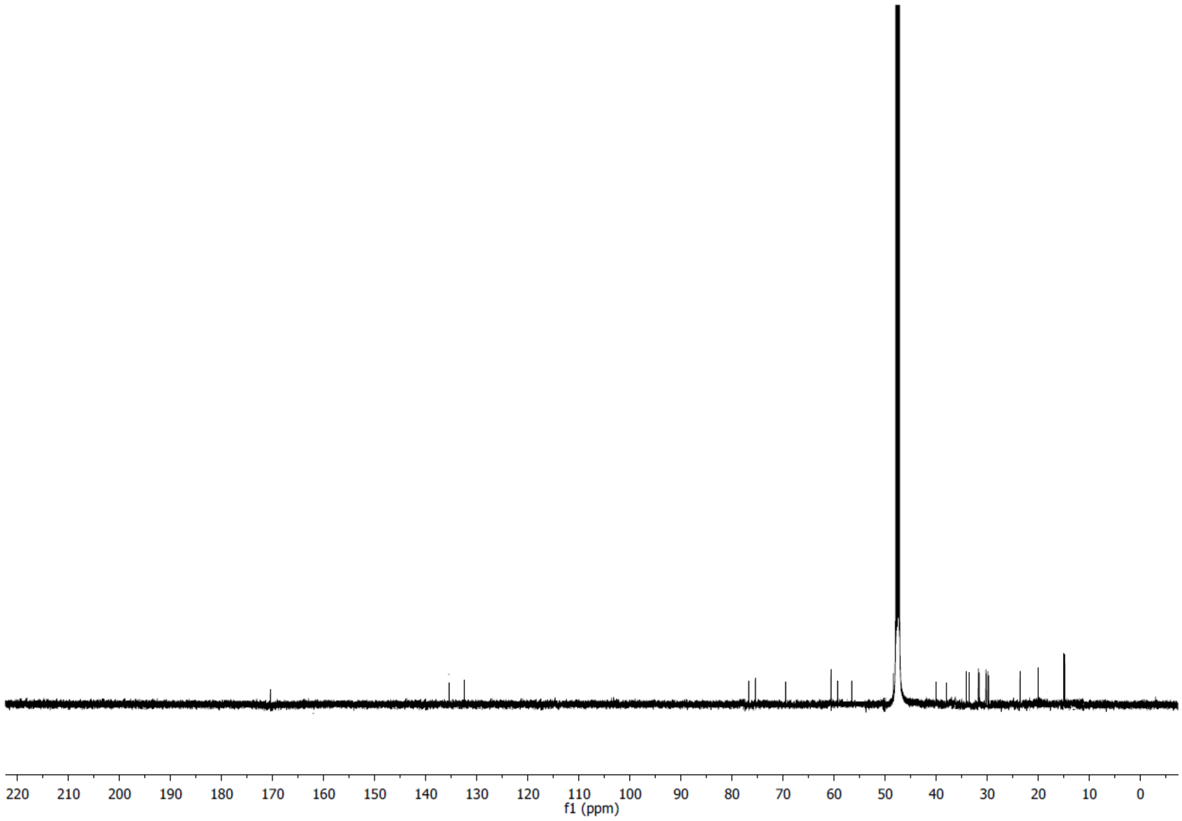

Figure S7. HR-ESIMS spectrum of lepadin L (**3**)

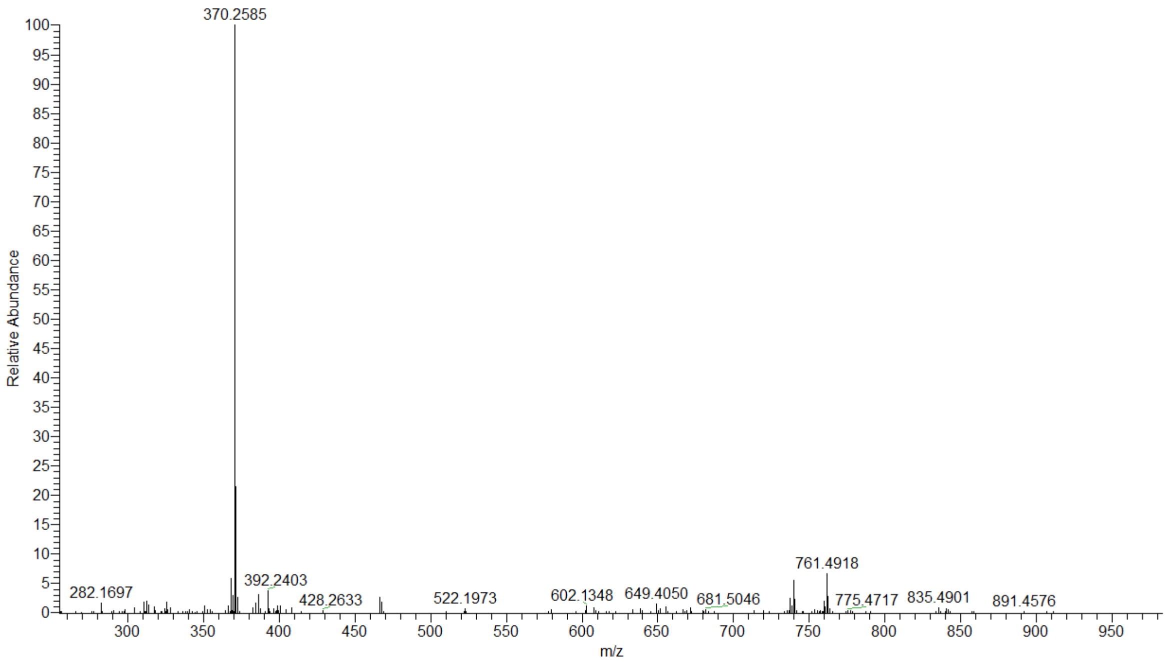

Figure S8. HR-ESIMS/MS spectrum of lepadin L (**3**)

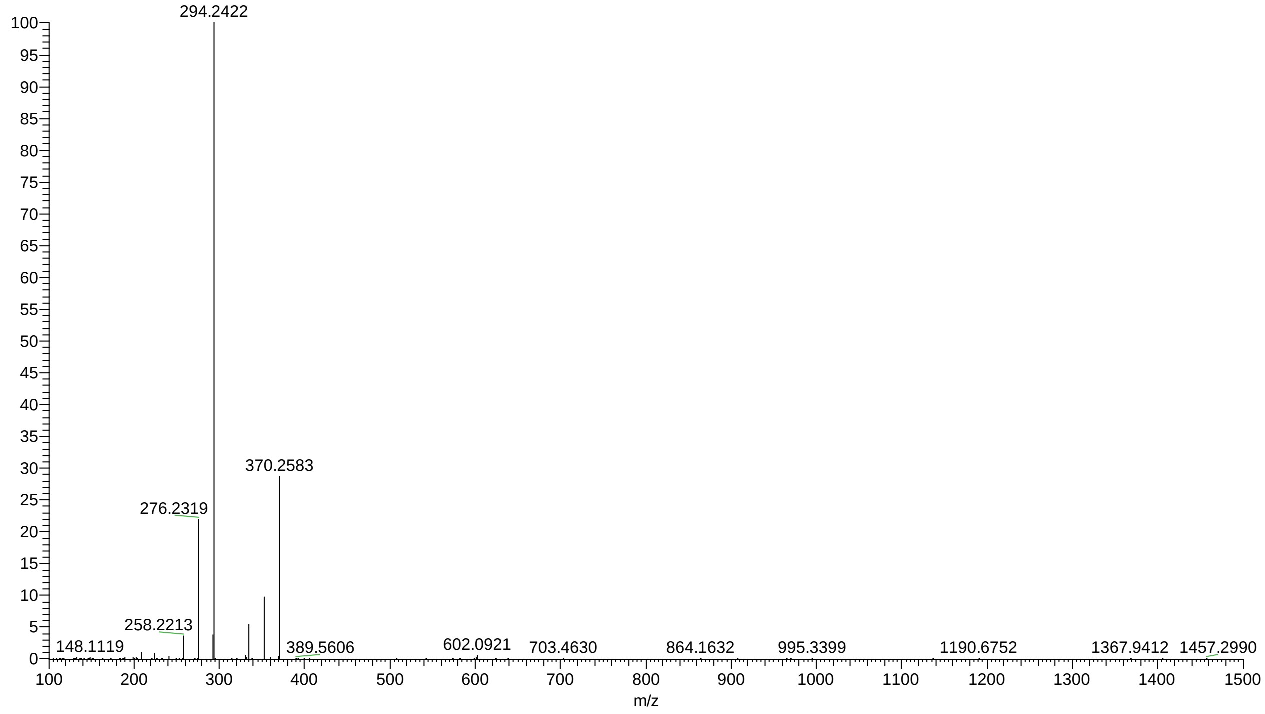

Figure S9. ^{1}H-^{1}H COSY NMR spectrum in CD_3OD (700 MHz) of lepadin L (**3**)

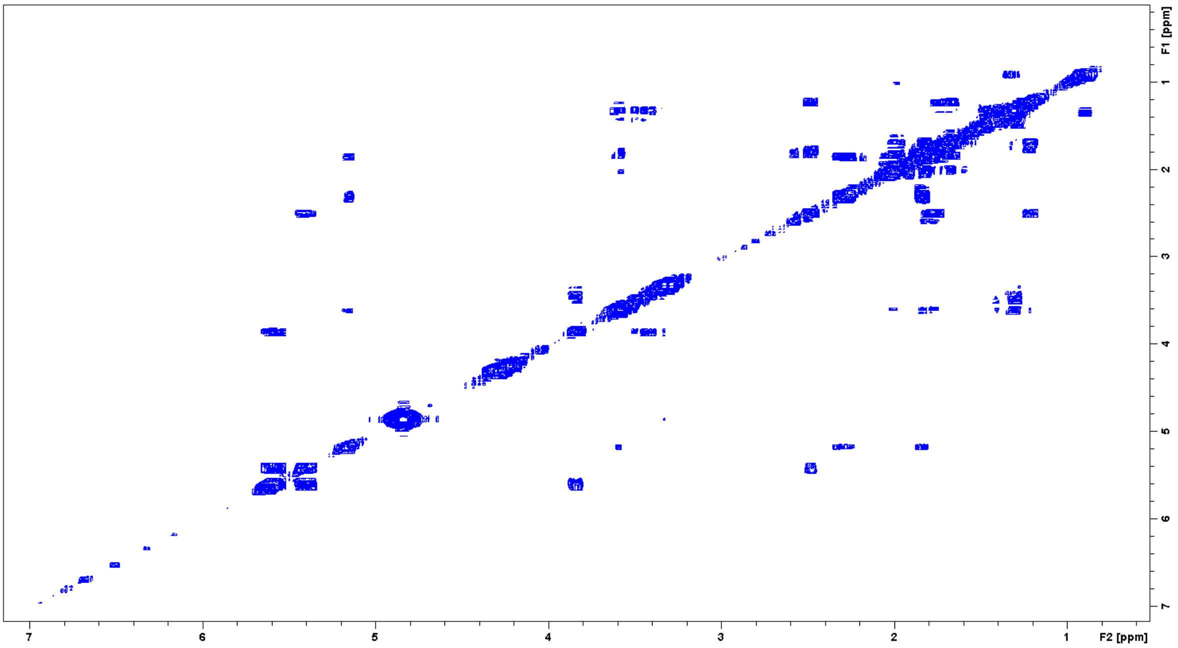

Figure S10. ^{1}H-^{13}C HSQC NMR spectrum in CD_3OD (700 MHz) of lepadin L (**3**)

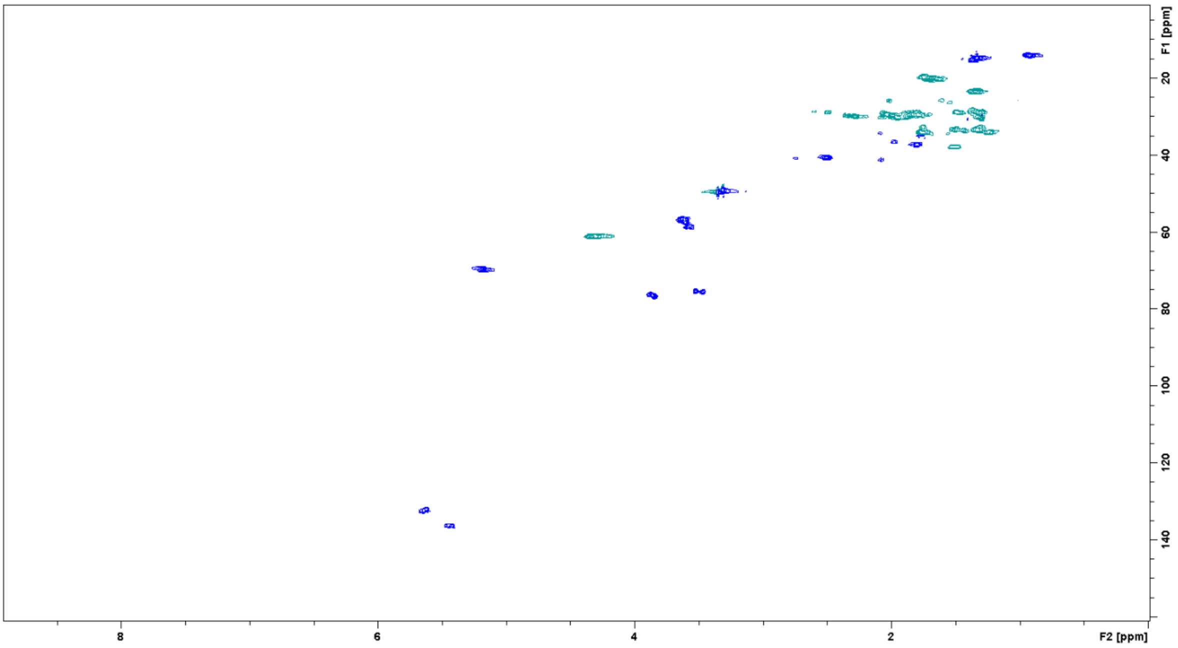

Figure S11. ^{1}H-^{13}C HMBC NMR spectrum in CD_3OD (700 MHz) of lepadin L (**3**)

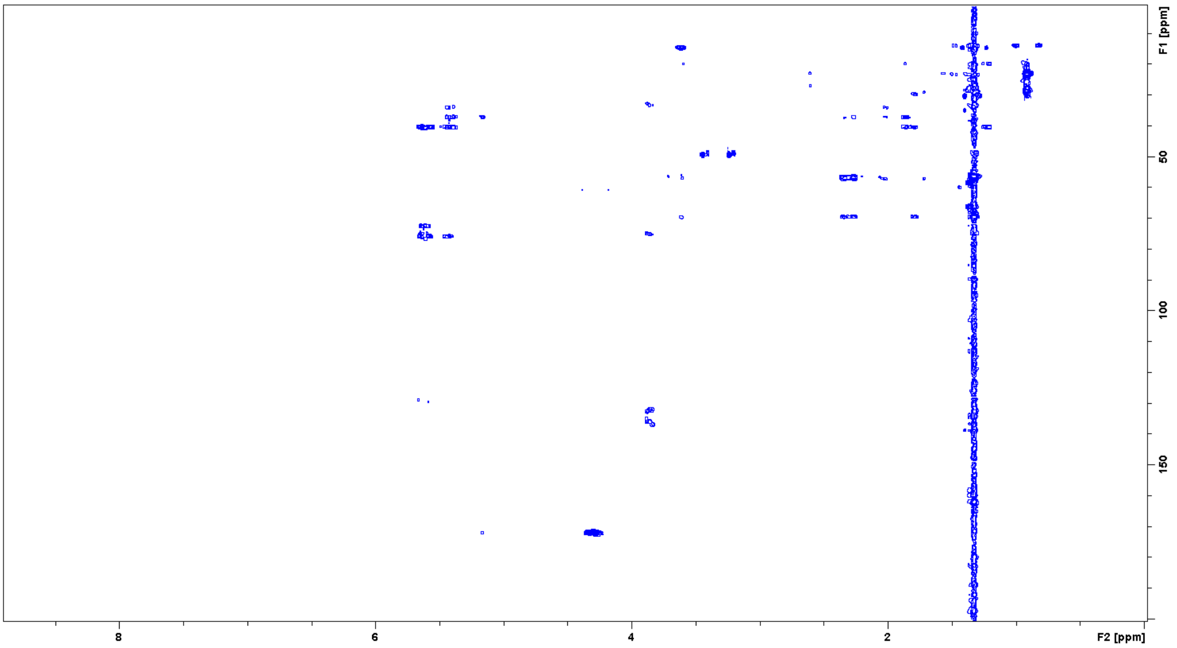

Figure S12. ^{1}H-^{1}H NOESY NMR spectrum in CD_3OD (700 MHz) of lepadin L (**3**)

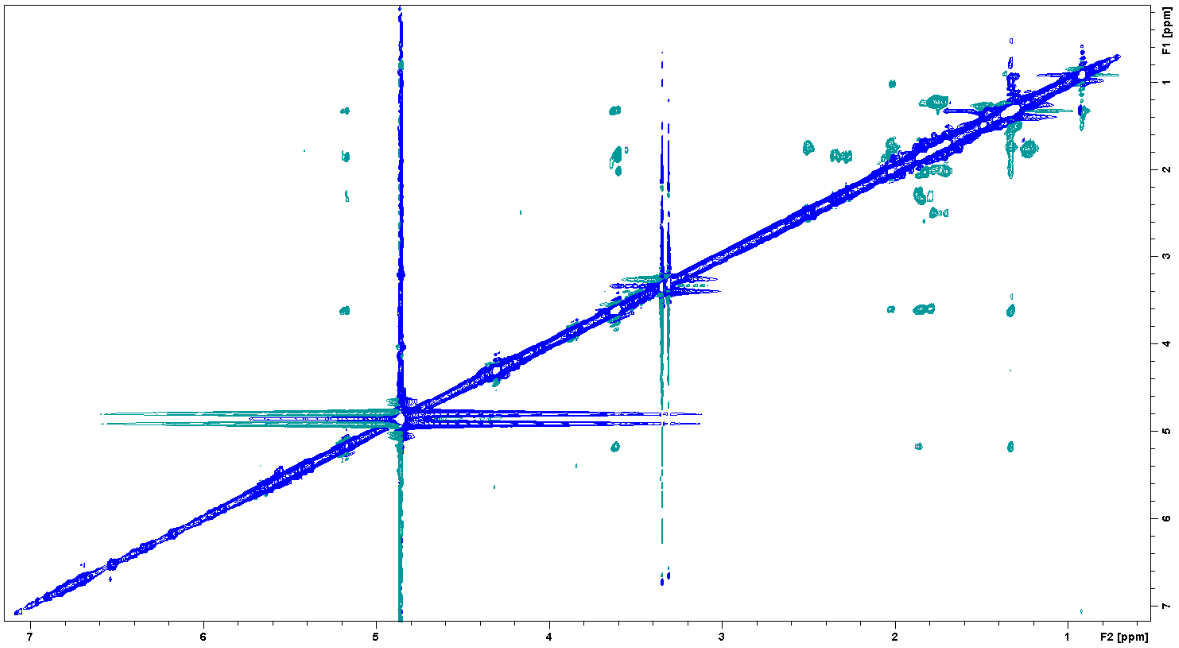

Figure S13. ^{1}H NMR spectrum in CD_3OD (700 MHz) of lepadin L acetonide (**4**)

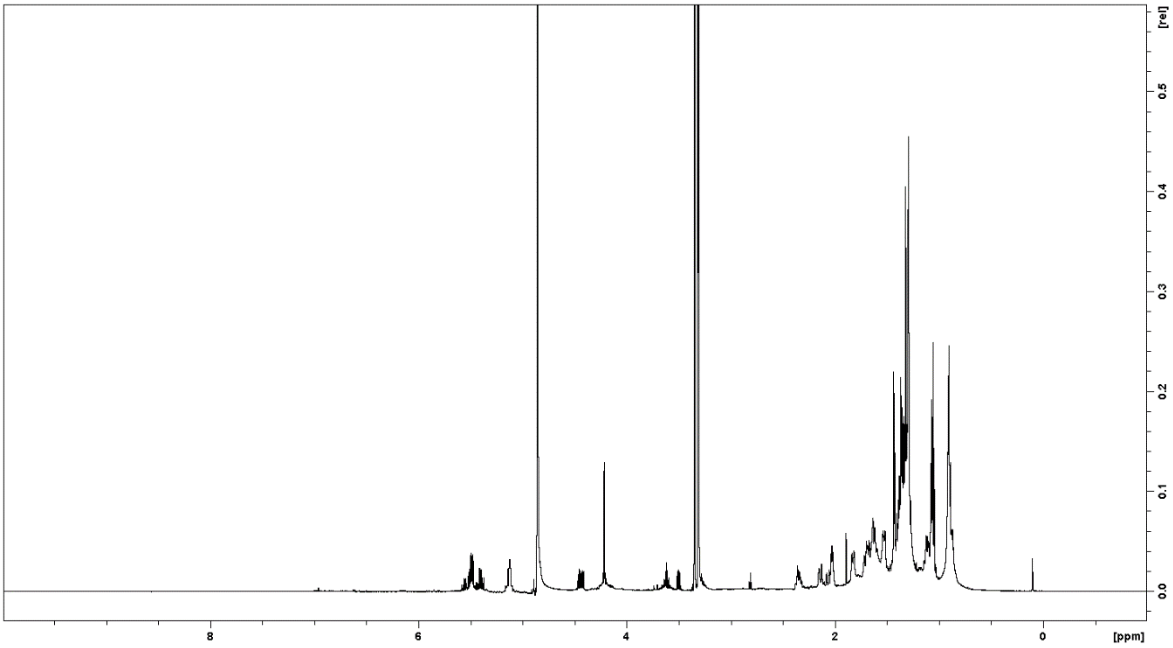

Figure S14. Enlargement of ^{1}H-^{13}C-HMBC spectrum in CD_3OD (700 MHz) of lepadin L acetonide (**4**)

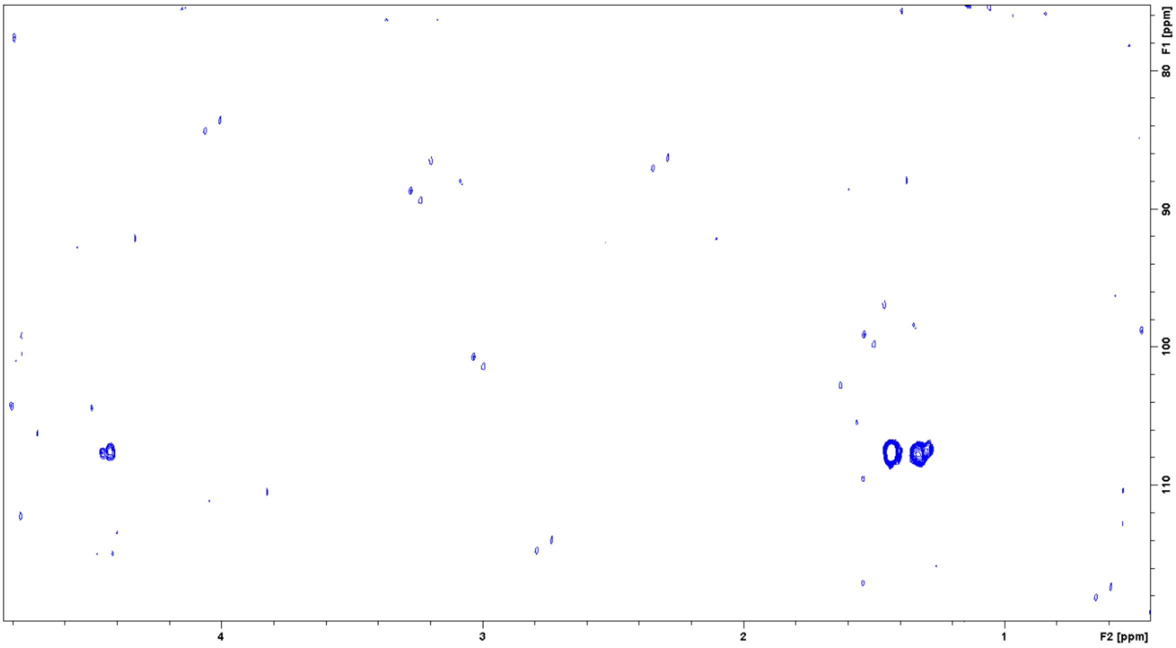

Figure S15. HR-ESIMS spectrum of lepadin L acetonide (**4**)

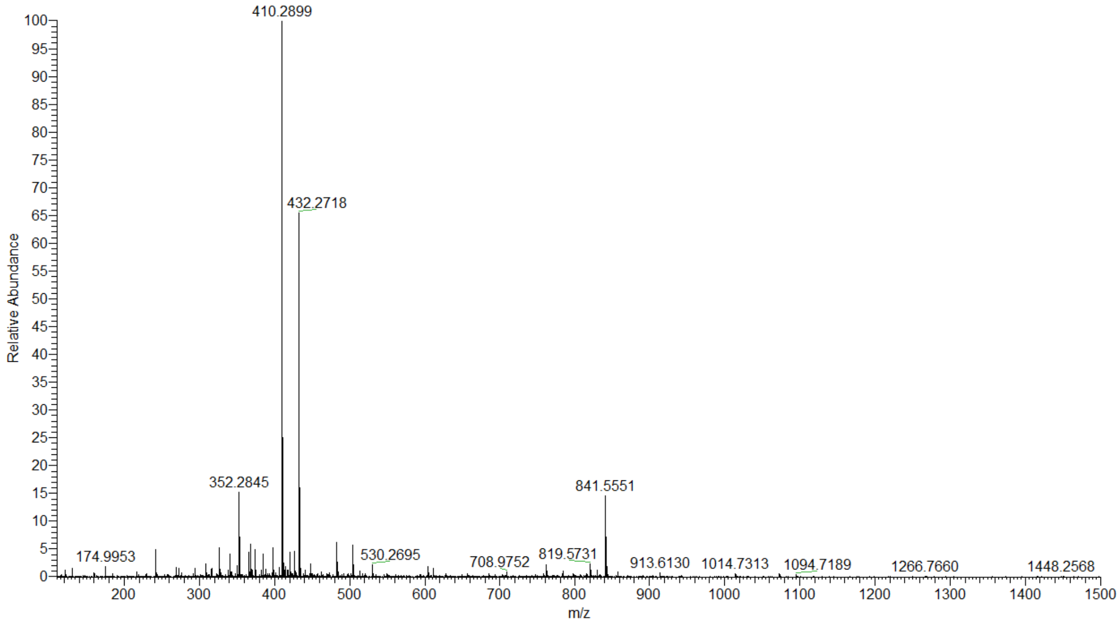

Supplement: Supplementary file 1 [file marinedrugs-20-00065-s001.zip › marinedrugs-1543072-supplementary.pdf]
